# Recruitment of mitofusin 2 into “lipid rafts” drives mitochondria fusion induced by Mdivi-1

**DOI:** 10.18632/oncotarget.24792

**Published:** 2018-04-10

**Authors:** Laura Ciarlo, Rosa Vona, Valeria Manganelli, Lucrezia Gambardella, Carla Raggi, Matteo Marconi, Walter Malorni, Maurizio Sorice, Tina Garofalo, Paola Matarrese

**Affiliations:** ^1^ Oncology Unit, Center for Gender-Specific Medicine, Istituto Superiore di Sanità, Rome, Italy; ^2^ Department of Experimental Medicine, Sapienza University, Rome, Italy; ^3^ Center for Behavioral Sciences and Mental Health, Istituto Superiore di Sanità, Rome, Italy; ^4^ Center of Metabolomics, Rome, Italy

**Keywords:** lipid rafts, mitochondria, fusion, apoptosis, reactive oxygen species

## Abstract

The regulation of the mitochondrial dynamics and the balance between fusion and fission processes are crucial for the health and fate of the cell. Mitochondrial fusion and fission machinery is controlled by key proteins such as mitofusins, OPA-1 and several further molecules. In the present work we investigated the implication of lipid rafts in mitochondrial fusion induced by Mdivi-1. Our results underscore the possible implication of lipid “rafts” in mitochondrial morphogenetic changes and their homeostasis.

## INTRODUCTION

Mitochondria are a highly responsive network of organelles in constant balance between two states: a set of individual spherical organelle or an elaborate network of interconnected elements [1]. The mitochondrial dynamics describe the continuous changes in the position, size and shape of mitochondria within cells [2], consisting in a balance of fusion and fission pathways, with each process mediated by a distinct set of interacting factors. This balance is of great relevance in both cell life and death since the mitochondrial network is involved in all the main cell activities such as metabolism, proliferation, autophagy and apoptosis [3]. Mitochondrial fusion machinery is controlled by three guanosine triphosphate (GTPase) proteins: mitofusin 1 (MFN1) and mitofusin 2 (MFN2) that mediate fusion of outer membrane, and optic atrophy protein 1 (OPA1) that carried out fusion of inner membrane [4–5]. The opposite process, mitochondria fission, is mediated, among others, by the dynamin-like protein 1 (DLP1), also called dynamin-related protein-1 (DRP1), which is recruited to the mitochondrial outer membrane, and, in human cells, by the human homologue of mitochondrial fission protein (hFIS1), initially described in yeast [6]. Interaction of DLP1 with hFIS1 [7] and mitochondrial fission factor (MFF1), a further molecule involved in the regulation of DRP1 activity [8], forms an oligomeric ring dividing mitochondria at distinct sites. The balance between fission and fusion is very important for mitochondrial participation in crucial cellular processes: fission is required for mitosis [9], apoptosis [10], and autophagy [11], while fusion is a necessary adaptation to nutrient starvation and increased metabolic demand [12] allowing transmission of transmembrane potential along interconnected mitochondria [13]. Disruption of mitochondrial dynamics is emerging as a pathogenetic determinant in prevalent diseases [14–15].

Since mitochondria fusion and fission are strictly related, the inhibition of one of these processes leads to an increase of the other: for instance, the inhibition of DLP1-mediated fission causes unopposed mitochondrial fusion [16]. In fact, 3-(2,4-Dichloro-5-methoxyphenyl)-2,3-dihydro-2-thioxo-4(1H)-quinazolinone (Mdivi-1), a selective inhibitor of mitochondrial fission that acts by inhibiting the GTPase activity of DLP1, actually induces mitochondrial fusion in a well-organized network [17]. Lipid rafts are membrane microdomains enriched in cholesterol and sphingolipids, which play many important roles in cell signal transduction. The structure of lipid rafts is dynamic, resulting in an ever-changing content of both lipids and proteins [18]. These structural platforms are not confined to the plasma membrane, but are similarly formed at subcellular organelles, which include endoplasmic reticulum, Golgi and mitochondria, named raft-like microdomains [18], or more shortly, “lipid rafts”. In previous works we have shown the pivotal role of these microdomains in fission machinery since the function of some proteins involved in mitochondria fission, such as DLP1, could be restricted to their localization in these structures [19–20]. In the present work we analyzed in some detail the possible implication of the rafts-like microdomains in the fusion process. We actually found that the disruption of microdomains impairs mitochondrial network formation and that the embedding of MFN2 in “lipid rafts” could be an essential event in the mitochondrial network extension induced by Mdivi-1.

## RESULTS

### Features of mitochondrial network formation induced by Mdivi-1

**Figure 1:**
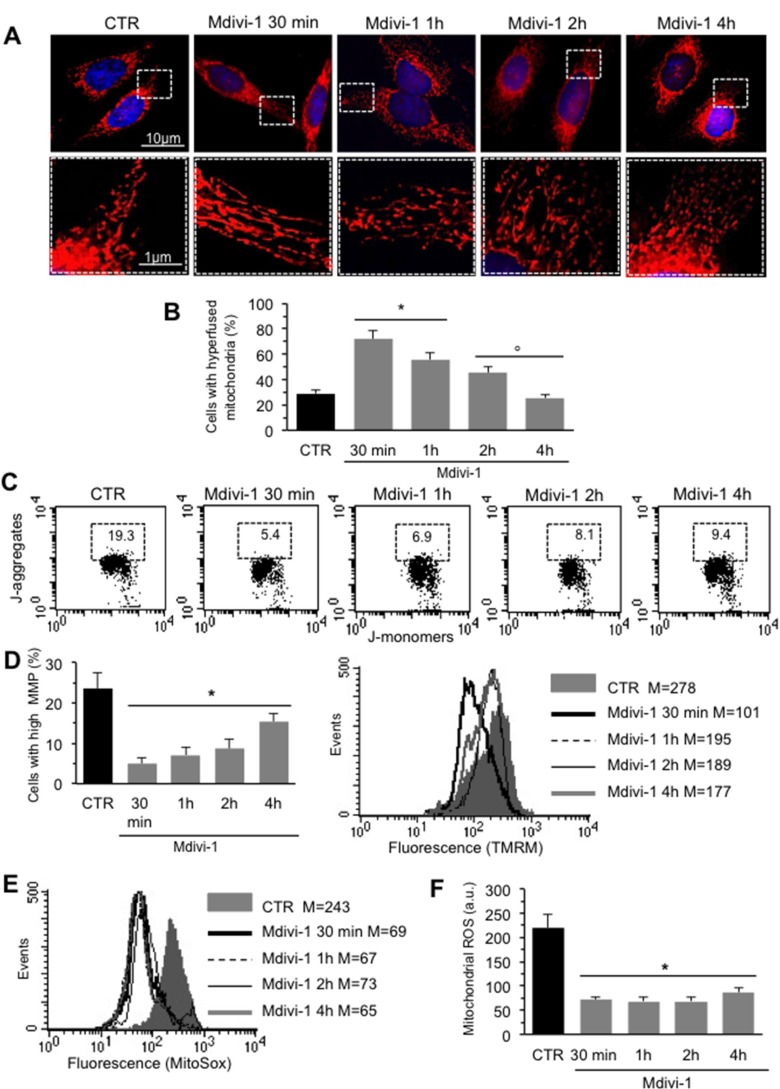
Mitochondrial network development induced by Mdivi-1 (**A**) *Upper line*. Time course IVM analysis of mitochondrial network of HeLa cells treated at different time points with Mdivi-1 after staining with anti-mitochondrion (red) and counterstaining with Hoechst (blue). *Bottom line*. Magnification of the boxed areas of the pictures shown in upper line. (**B**) Bar graph showing morphometric analysis results. In ordinate the percentage of cells with elongated mitochondria. Data are reported as mean value ± SD of the results obtained in three independent experiments. ^*^p < 0.01 *vs* CTR; °*p <* 0.01 *vs* Mdivi-1 30 min. (**C**) Representative dot plots of the cytofluorimetric analysis of MMP, performed by using JC-1, of HeLa cells treated at different time points with Mdivi-1. Numbers in the boxed areas indicate the percentage of cells with high MMP. (**D**) *Left panel*. Bar graph showing the results of the analysis of MMP obtained from three independent experiments and reported as mean ± SD. ^*^p < 0.01 *vs* CTR. *Right panel.* Representative cytofluorimetric histograms illustrating results obtained by using TMRM in HeLa cells treated at different time points with Mdivi-1. Numbers indicate the median fluorescence intensity. (**E**) Flow cytometric analysis, performed by using MitoSOX-red, of mitochondrial ROS production of a representative experiments after cell treatment with Mdivi-1 at different time points. Numbers represent the median fluorescence intensity. (**F**) Bar graph showing the results of the analysis of mitochondrial ROS production obtained in three independent experiments and reported as mean ± SD of the median fluorescence intensity. ^*^p < 0.01 *vs* CTR.

**Figure 2:**
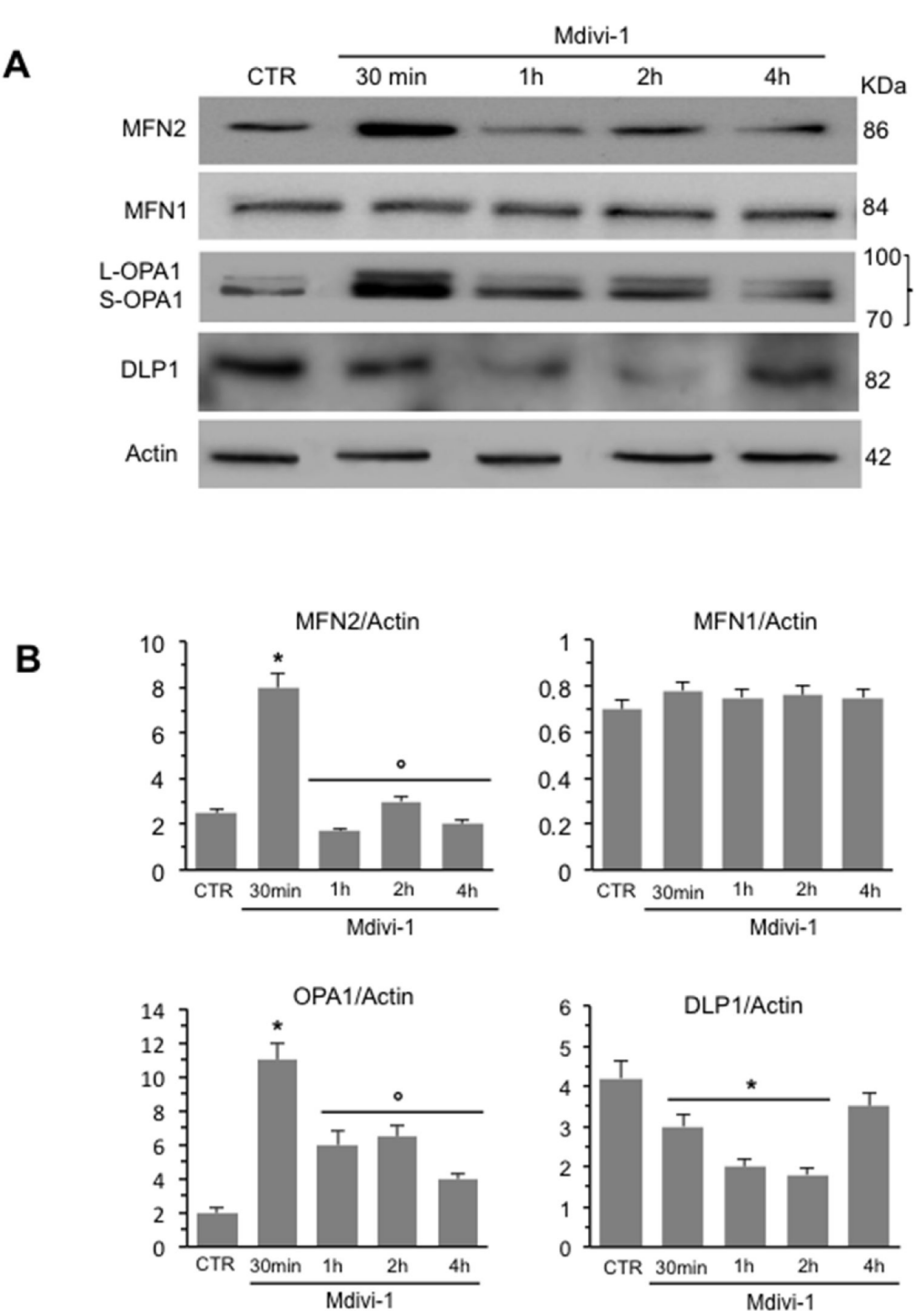
Time course analysis of the expression of key proteins in mitochondria dynamics (**A**) Western blot analysis of MFN2, MFN1, OPA1 and DLP1 of HeLa cells treated at different time points with Mdivi-1. (**B**) Bar graphs of densitometric analysis of western blot signals expressed as arbitrary units (a.u.). To note, after 30 min of Mdivi-1 triggering, the increase of MFN2 and OPA1 and decrease of DLP1. ^*^p < 0.01 *vs* CTR; °*p <* 0.01 *vs* Mdivi-1 30 min. No statistically significant differences of MFN1 expression levels was evident after Mdivi-1 administration.

We analyzed mitochondrial network development process in HeLa cells by using Mdivi-1, a selective inhibitor of mitochondrial division that acts by inhibiting the GTPase activity of DLP1. First of all, we performed a time course analysis of mitochondrial network organization by immunofluorescence microscopy after staining with anti-mitochondrion (red) and counterstaining with Hoechst (blue) of cells treated at different time points (30 min, 1 hour, 2 hours and 4 hours) with Mdivi-1. We found that early after Mdivi-1 administration (30 min) mitochondrial network became more organized and characterized by fused elongated structures to form a reticulum in comparison to untreated control cells (Figure 1A, upper and bottom line, compare second panel with first panel). This improvement of mitochondrial network organization became less evident with time (Figure 1A, upper and bottom line, compare second panel with third, fourth and fifth panels). In fact, as demonstrated by morphometric analysis (Figure 1B), mitochondrial elongation process peaked 30 min after Mdivi-1 administration and then “slowly” reverted to normal values (1, 2 and 4 hours after treatment). In particular, in cells treated with Mdivi-1 for 30 min the percentage of cells showing “hyperfused” mitochondria was significantly (*p <* 0.01) higher in respect either to control cells or to cells treated with Mdivi-1 for 1, 2 and 4 hours (Figure 1B). The analysis of mitochondrial membrane potential performed by flow cytometry, after cell staining with JC-1, revealed that Mdivi-1 significantly reduced (*p <* 0.01) the percentage of cells with high mitochondrial membrane potential (boxed area in Figure 1C) detectable in control cells. This effect was evident starting from 30 min after treatment and persisted up to 2 hours (dot plots in Figure 1C show a representative experiment and bar graph in Figure 1D shows results obtained from four independent experiments). High mitochondrial membrane potential was previously associated either with cell “sensitization” to apoptosis induction or with high ROS generation [21–22]. In line with this, we found that treatment with Mdivi-1 also significantly reduced mitochondrial ROS generation (Figure 1E shows a representative experiment and bar graph in Figure 1F reports data obtained from four independent experiments). We then analyzed by western blot some key proteins involved in mitochondrial fusion process: MFN2, MFN1 and OPA1. We observed a significant increase of the expression levels of both MFN2 and OPA1 30 min after Mdivi-1 administration. In particular, the analysis of OPA1 expression revealed a significant increase in both longer and shorter forms, confirming that a specific combination of the two forms is also required for efficient mitochondrial fusion in Hela cells [23]. After this time, the amount of these proteins decreased progressively. Furthermore, to exclude an involvement of MFN1 in mitochondrial fusion process, since it is known to be quite similar to MFN2 both in primary sequence and in secondary structure, we analyzed MFN1 expression levels under the same experimental conditions. As showed in Figure 2A, no difference of MFN1 expression levels was evident after Mdivi-1 administration. On the contrary, a reduction of DLP1, a key protein in the mitochondrial fission processes, was observed 30 min after Mdivi-1 administration in comparison to control cells (Figure 2A). These results were also confirmed by densitometric analysis performed pooling together data obtained from three independent experiments (Figure 2B).

### Involvement of “lipid rafts” in mitochondrial network organization

**Figure 3:**
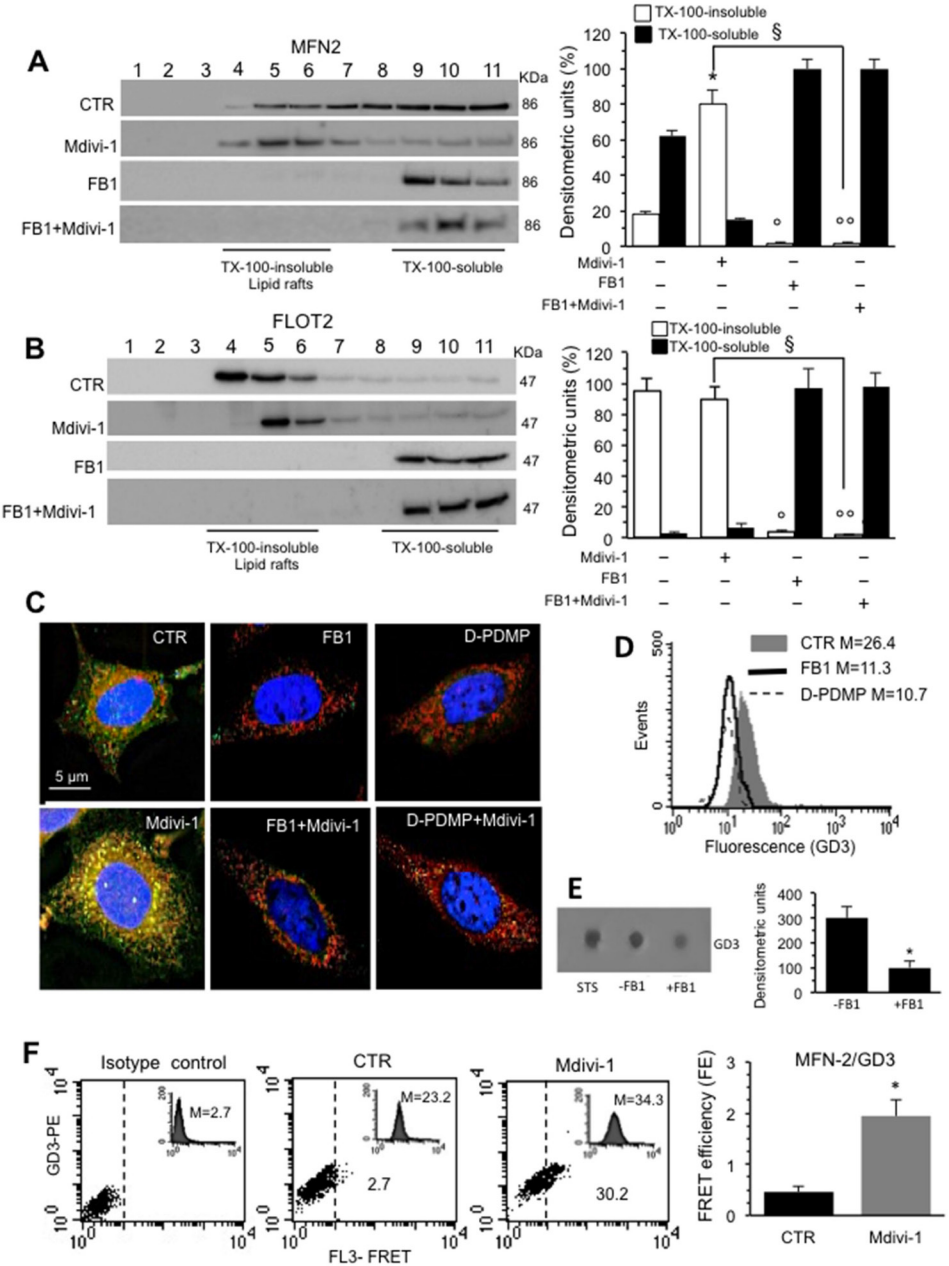
Involvement of lipid rafts in mitochondrial network organization: analysis of MFN2/GD3 association (**A**) *Left panel.* Western blot analysis of sucrose gradient fractions of HeLa cells untreated or treated with Mdivi-1 and FB1 (alone or in combination). To note, MFN2 was essentially distributed in fractions 5–11 in untreated control cells. After 30 min of Mdivi-1 triggering, MFN2 was enriched in fractions 4-6, corresponding to raft-like microdomains. In cells treated with FB1, alone or in combination with Mdivi-1, MFN2 was completely absent in fractions 4-6. *Right panel*. Densitometric analyses performed on three independent experiments. (**B**) *Left panel*. Analyses of the distribution of the FLOT2 by sucrose gradient fractions. (A, B) *Right panels.* Bar graphs of the densitometric analysis. The columns indicate the percent distribution across the gel of fractions 4 to 6 (Triton X-100-insoluble fractions) and 9 to 11 (Triton X-100-soluble fractions), as detected by densitometric scanning analysis. Results represent the mean ± SD from three independent experiments. ^*^p < 0.01, TX-100-insoluble Mdivi-1 *vs* CTR; °*p <* 0.01, TX-100-insoluble FB1 *vs* CTR; °°*p <* 0.01, TX-100-insoluble FB1+Mdivi-1 *vs* CTR; ^§^*p <* 0.01, TX-100-insoluble FB1+Mdivi-1 *vs* Mdivi-1. (**C**) Immunofluorescence analysis of HeLa cells, either untreated or treated with Mdivi-1 (30min), after staining with anti-GD3 (green fluorescence), anti-MFN2 (red fluorescence) and counterstaining with Hoechst (blue). To note, the yellow fluorescence (resulting from the overlay of green and red fluorescence) indicating the co-localization of GD3 and MFN2 after Mdivi-1 administration. (**D**) Cytofluorimetric histograms showing the amount of GD3 in HeLa cells untreated or treated with FB1 or D-PDMP obtained in a representative experiment. Cells treated with FB1 or D-PDMP showed a very low amount of GD3. Numbers indicate the median fluorescence intensity. (**E**) Dot blot analysis of GD3 from untreated or FB1 pre-treated cells. Results represent the mean ± SD from 3 independent experiments. *P* values were determined using ANOVA with Newman-Keuls multiple-comparison test. ^*^p < 0.01, FB1 pre-treated cells *vs* CTR. (**F**) Quantitative evaluation of MFN2/GD3 association by FRET technique by flow cytometry analysis. Bar graph represents the FRET efficiency and indicate that MFN2/GD3 association was significantly higher in Mdivi-1 treated cells respect to untreated cells. ^*^p < 0.01 *vs* CTR.

We then examined the implication of “lipid rafts” in the processes described above. In these dynamic structures, also present in mitochondria and others organelles, some molecules, including gangliosides (GD3, GM3 etc.), cholesterol and the voltage-dependent anion channel-1 (VDAC-1), are constitutively present, whereas others, such as DLP1, can be recruited [20]. On the bases of the above results, we selected 30 min-treatment with Mdivi-1 to carry out biochemical analyses, focusing on MFN2, a mitochondrial membrane protein that participates in mitochondrial fusion in mammalian cells, contributing to the maintenance of the mitochondrial network and metabolism [24]. In particular, we analyzed the association of MFN2 both in Triton X-100- insoluble and -soluble fractions. As shown in Figure 3A (left panel), the analysis of the fractions obtained by a 5–30% linear sucrose gradient revealed that MFN2 was essentially distributed in fractions 5–11 in untreated control cells. After 30 min of Mdivi-1 triggering, MFN2 became enriched in fractions from 4 to 6, corresponding to raft microdomains. In cells treated with fumonisin B1 (FB1), an inhibitor of ceramide synthase that alters lipid raft composition and molecular organization, alone or in combination with Mdivi-1, we found that MFN2 was completely absent in fractions 4-6 corresponding to rafts microdomains (Figure 3A, left panel). As a methodological control, we also analyzed the distribution of flotillin 2 (FLOT2), a marker of lipid rafts, under the same experimental conditions. As expected, FLOT2 was found, highly enriched in the raft fractions 4–6 both in untreated and Mdivi-1-treated cells becoming completely absent in cells treated with FB1 alone or in combination with Mdivi-1 (Figure 3B, left panel). These results were also confirmed by densitometric analyses, performed pooling together data obtained from three independent experiments (Figure 3A and 3B, bar graph for MFN2 and for FLOT2 respectively, right panels). Moreover, fluorescence microscopy observations further confirmed these data. In fact, IVM analysis after triple staining with anti-GD3 antibodies (green), anti-MFN2 antibodies (red) and Hoechst dye (blue) revealed a significant co-localization of GD3 and MFN2 (see yellow fluorescent areas resulting from green and red fluorescence overlay) only in cells treated with Mdivi-1 (Figure 3C). Interestingly, cells treated with FB1 as well as with (+)- threo-1-phenyl-2-decanoylamino-3-morpholino-1-propanol hydrochloride ([D]-PDMP) (alone or in combination with Mdivi-1) showed a very low amount of GD3 and no co-localization with MFN2 was observed (Figure 3C). Similar results were obtained by using 5 mM methyl-β-cyclodextrin (MβCD) for 30 min at 37° C, which disrupts lipid rafts by removing cholesterol (Supplementary Figure 1A and 1B, which also shows pictures of the staining of the individual markers).

As a control, to check the GD3 content after FB1 or [D]-PDMP pre-treatment, either flow cytometry or dot-blot analysis was performed. As expected, both FB1 and [D]-PDMP caused a significant decrease in GD3 expression (Figure 3D, flow cytometry analysis) also confirmed by dot-plot and densitometric analysis of FB1 (Figure 3E) or [D]-PDMP treatment (data not shown), revealing that, both FB1 and [D]-PDMP pre-treatment are also able to affect ganglioside content (Figure 3D and 3E). Moreover, TLC analysis was performed to check the total cholesterol content in both untreated and MβCD treated cells (Supplementary Figure 1C).

Parallel FRET analysis indicated that MFN2/GD3 association was evident 30 min after Mdivi-1 administration (Figure 3F) and decreased progressively by time until 4 hours (data not shown), as also demonstrated by FRET efficiency (FE) evaluation (Figure 3F, bar graph) performed according to the study by Riemann and collaborators [25].

**Figure 4:**
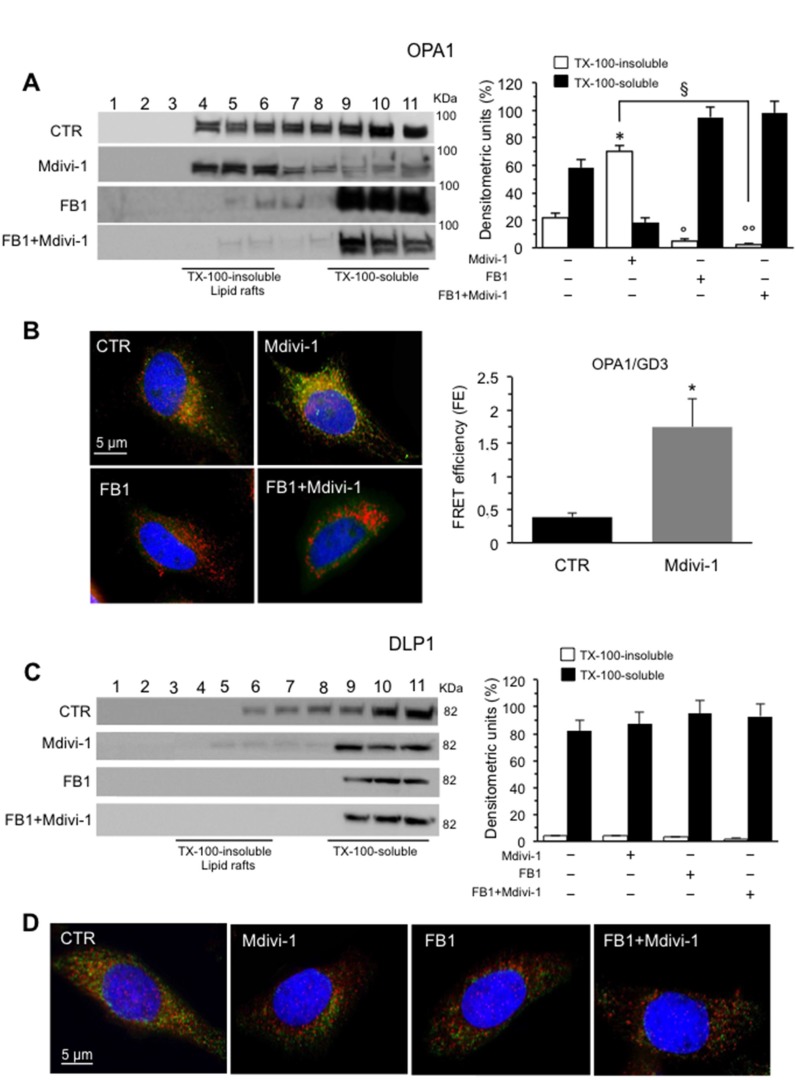
Involvement of “lipid rafts” in mitochondrial network organization: analysis of OPA1/GD3 and DLP1/GD3 association (**A**) *Left panel.* Western blot analysis of sucrose gradient fractions of HeLa cells untreated or treated with Mdivi-1 and FB1 (alone or in combination). To note, OPA1 was essentially distributed in fractions 4–11 in untreated control cells (CTR). After 30 min of Mdivi-1 triggering, OPA1 was enriched in lipid rafts (fractions 4 and 6). In cells treated with FB1, alone or in combination with Mdivi-1, OPA1 was almost absent in fractions 4-6. *Right panel*. Bar graph of densitometric analysis of sucrose gradient fractions. The columns indicate the percent distribution across the gel of fractions 4-5-6 (Triton X-100-insoluble fractions) and 9-10-11 (Triton X-100-soluble fractions), as detected by densitometric scanning analysis. Results represent the mean ± SD from three independent experiments. ^*^p < 0.01, TX-100-insoluble Mdivi-1 *vs* CTR; °*p <* 0.01, TX-100-insoluble FB1 *vs* CTR; °°*p <* 0.01, TX-100-insoluble FB1+Mdivi-1 *vs* CTR; ^§^*p <* 0.01, TX-100-insoluble FB1+Mdivi-1 *vs* Mdivi-1. (**B**) Immunofluorescence analysis of HeLa cells, either untreated or treated with Mdivi-1 (30 min), after staining with anti-GD3 (green fluorescence), anti-OPA1 (red fluorescence) and counterstaining with Hoechst (blue). To note, yellow fluorescence (resulting from the overlap of green and red fluorescence) indicating the co-localization of GD3 and OPA1 after Mdivi-1 triggering. Cells treated with FB1 (alone or in combination with Mdivi-1) showed a very low amount of GD3 and no co-localization with OPA1 was observed. (**C**) *Left panel.* Western blot analysis of sucrose gradient fractions of DLP1. To note, DLP1 was essentially distributed in fractions 6–11 in untreated control cells, but was completely absent in fractions 4-6, corresponding to lipid rafts, in Mdivi-1-treated cells. *Right panel*. Bar graph of densitometric analysis of sucrose gradient fractions. The columns indicate the percent distribution across the gel of fractions 4-6 (Triton X-100-insoluble fractions) and 9–11 (Triton X-100-soluble fractions), as detected by densitometric scanning analysis. Results represent the mean ± SD from three independent experiments. No statistically significant differences were found between Triton X-100-insoluble fractions from CTR cells and Triton X-100-insoluble fractions from Mdivi-1 treated cells. (**D**) Immunofluorescence analysis of HeLa cells, either untreated or treated with Mdivi-1, after staining with anti-GD3 (green fluorescence), anti-DLP1 (red fluorescence) and counterstaining with Hoechst (blue). To note, yellow fluorescence, indicating the co-localization of GD3 and DLP1, only in untreated control cells.

To go deep inside the mitochondrial dynamics, we also analyzed OPA1 and DLP1 in fractions obtained by a 5–30% linear sucrose gradient. We observed that in control cells OPA1 was present in fractions 4–6, corresponding to “lipid rafts”, as well as in fractions 9-11 corresponding to soluble fractions. By contrast, after Mdivi-1 administration, it was detected exclusively in the detergent-insoluble fractions 4–6, indicating that OPA1 was recruited into raft-like fractions after treatment with Mdivi-1 (Figure 4A, left panel). In cells treated with FB1, alone or in combination with Mdivi-1, we noted an evident decrease in the amount of OPA1 from fractions 4-6 which was detectable mostly in fractions 9-11, as also shown by densitometric analyses performed pooling together data obtained from three independent experiments (Figure 4A, right panel). These results were also confirmed both by fluorescence (Figure 4B, left panels, see Supplementary Figure 2 for staining of the individual markers) and FRET analyses by calculation of FE by using Riemann’s algorithm (Figure 4B, right panel) that revealed a significant co-localization OPA1/GD3 in Mdivi-1 treated cells only. Similar results were obtained by MβCD (data not shown).

As far as DLP1 was concerned, we found that in control cells it was distributed in fractions 6–11 appearing almost detectable at soluble fractions 10-11, as previously reported by us [20]. After treatment with Mdivi-1 or FB1, alone or in combination, DLP1 was completely restricted at detergent-soluble fractions 9-11. (Figure 4C, left panel) as also shown by densitometric analyses (Figure 4C, bar graph right panel). In fact, as shown by fluorescence analyses, a weak physiological association of DLP1 with GD3 observed in untreated control cells was strongly reduced after cell treatment with Mdivi-1 (Figure 4D, see Supplementary Figure 3 for staining of the individual markers). This is not surprising, since DLP1 appears to be recruited to mitochondria to mediate fission activities [6, 26].

### Effect of ganglioside depletion by FB1 in isolated mitochondria

**Figure 5:**
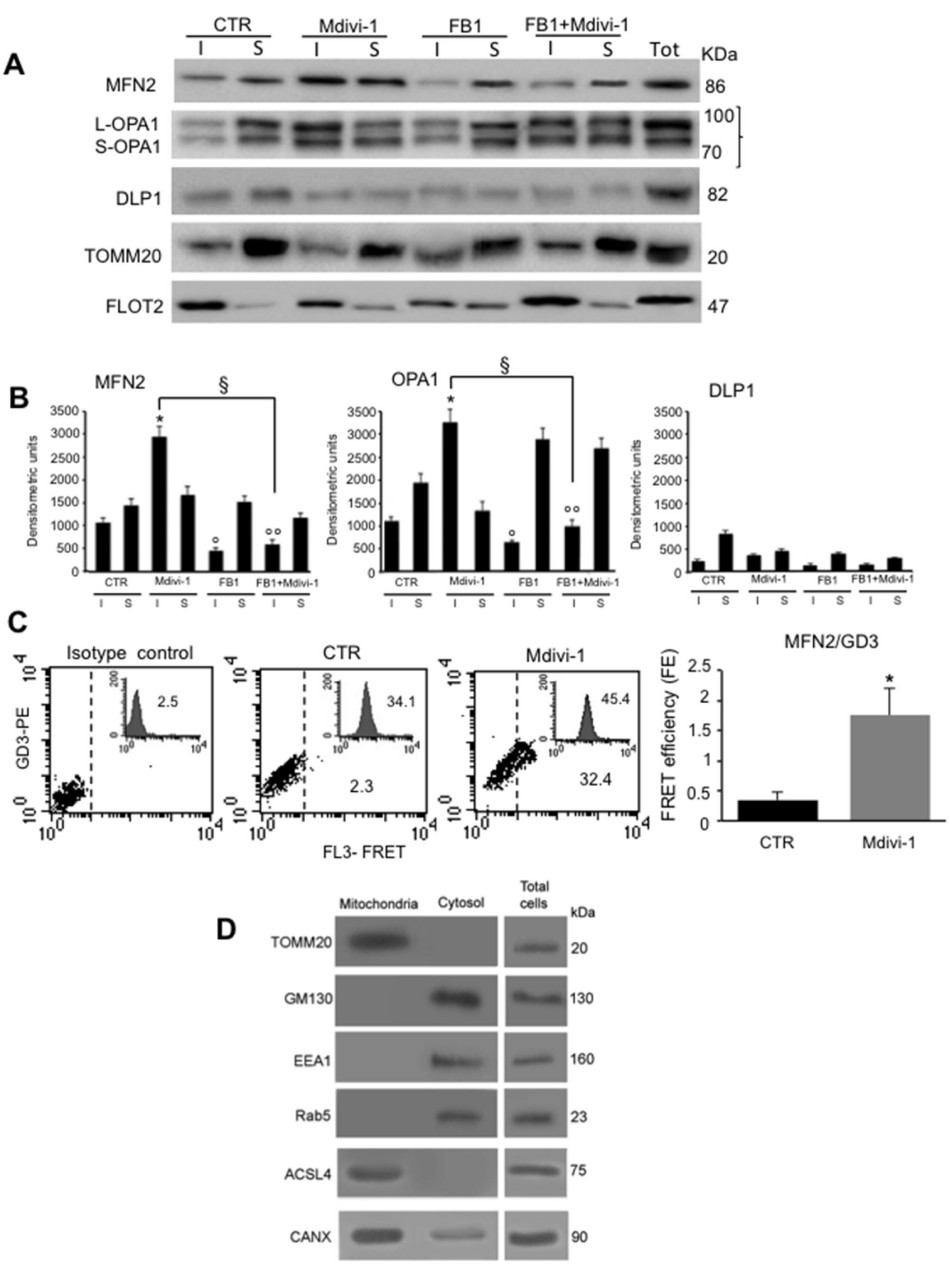
Effect of ganglioside depletion by FB1 in isolated mitochondria (**A**) Isolated mitochondria from HeLa cells, either untreated or treated with Mdivi-1 (30 min) or FB1 (alone or in combination). Both Triton X-100-insoluble (I) and soluble (S) fractions were analyzed by western blot analysis and probed with anti-MFN2, anti-OPA1 and anti-DLP1. To note, an increase of MFN2 and OPA in both detergent-insoluble and soluble fractions after Mdivi-1 stimulation. As a control, we also analyzed the distribution of the TOMM20 and FLOT2. The fraction samples were loaded by volume. (**B**) Bar graphs indicate densitometric analysis of (I) and (S) fractions. Results represent the mean ± SD from three independent experiments. ^*^p < 0.01, insoluble fractions from Mdivi-1 *vs* CTR; °*p <* 0.01, insoluble fractions from FB1 *vs* CTR; °°*p <* 0.01, insoluble fractions from FB1+Mdivi-1 *vs* CTR; ^§^*p <* 0.01, insoluble fractions from FB1+Mdivi-1 *vs* Mdivi-1; no statistically significant differences of DLP1 expression levels was evident after Mdivi-1 or FB1+Mdivi-1 administration. (**C**) Quantitative evaluation of MFN2/GD3 association by FRET technique, as revealed by flow cytometry analysis in intact isolated mitochondria. Bar graph represents the FRET efficiency, which indicates that MFN2/GD3 association was significantly higher in Mdivi-1 treated cells respect to untreated cells. ^*^p < 0.01 *vs* CTR. (**D**) Western blot analyses to check the purity of mitochondria preparations.

To confirm that proteins involved in mitochondrial network formation were present in raft-like microdomains at mitochondrial level, we performed the same analysis both in Triton X-100-insoluble and -soluble fractions obtained from isolated mitochondria (Figure 5A, upper panel). In untreated control cells we found MFN2 and OPA1 either in the detergent-soluble or insoluble fractions. Conversely, after Mdivi-1 stimulation, an increase of MFN2 and OPA1 in Triton X-100- insoluble fractions, was observed. After treatment with FB1, alone or in combination with Mdivi-1, these proteins were essentially present in the soluble fraction, similarly to what observed in control cells. By contrast, DLP1 showed a preferential localization in Triton X-100-soluble fractions respect to Triton X-100-insoluble fractions obtained from control cells, remaining virtually the same after treatment with Mdivi-1 and FB1 (alone or in association). According to literature data, we found that TOMM20, a typical mitochondrial marker used as a control, was present in detergent-soluble, but not in detergent-insoluble fractions. On the contrary, as expected, FLOT2 was found highly enriched in Triton X-100-insoluble fraction respect to Triton X-100-soluble fraction both in untreated and Mdivi-1-treated cells, decreasing in Triton X-100-insoluble fraction of cells treated with FB1. These results were also confirmed by densitometric analyses, performed pooling together data obtained from three independent experiments (Figure 5B, bar graph). Biochemical analyses were paralleled by FRET evaluation performed in intact isolated mitochondria in order to assess possible molecular interaction of MFN2 and GD3 ganglioside, selected as paradigmatic component of mitochondrial raft-like microdomains. Quantitative FRET analysis of mitochondria isolated from control cells revealed a minimal molecular interaction between GD3 and MFN2 (Figure 5C, second panel. Compare with isotype control in first panel). The association of GD3 with MFN2 became significant after treatment with Mdivi-1 (Figure 5C, third panel), as also demonstrated by calculation of FE by using Riemann’s algorithm (Figure 5C, bar graph).

The purity of our crude mitochondria preparations was checked by western blot (Figure 5D) by analyzing the mitochondrial marker TOMM20, anti-early endosome antigen 1 (EEA1), the endolysosomal antigen Ras-related protein 5 (Rab5), the Golgi marker 130 (GM130). In addition, the key molecular marker CANX, known to be enriched in the MAMs [27] and ACSL4, (FOCL4) a single-pass membrane protein localizing to the mitochondrion, microsome or peroxisome, were checked by western blot analysis. Altogether, biochemical analyses showed that mitochondrial preparation was virtually pure since it was negative either to endosomal or Golgi apparatus specific markers, although the presence of ACSL4 and CANX markers demonstrated the presence of MAMs in these fractions, suggesting a possible participation of MAM in the regulation of mitochondrial fusion. As expected, mitochondrial fractions were positive to TOMM20 (Figure 5D).

### Effect of MF2 silencing on mitochondria network organization

**Figure 6:**
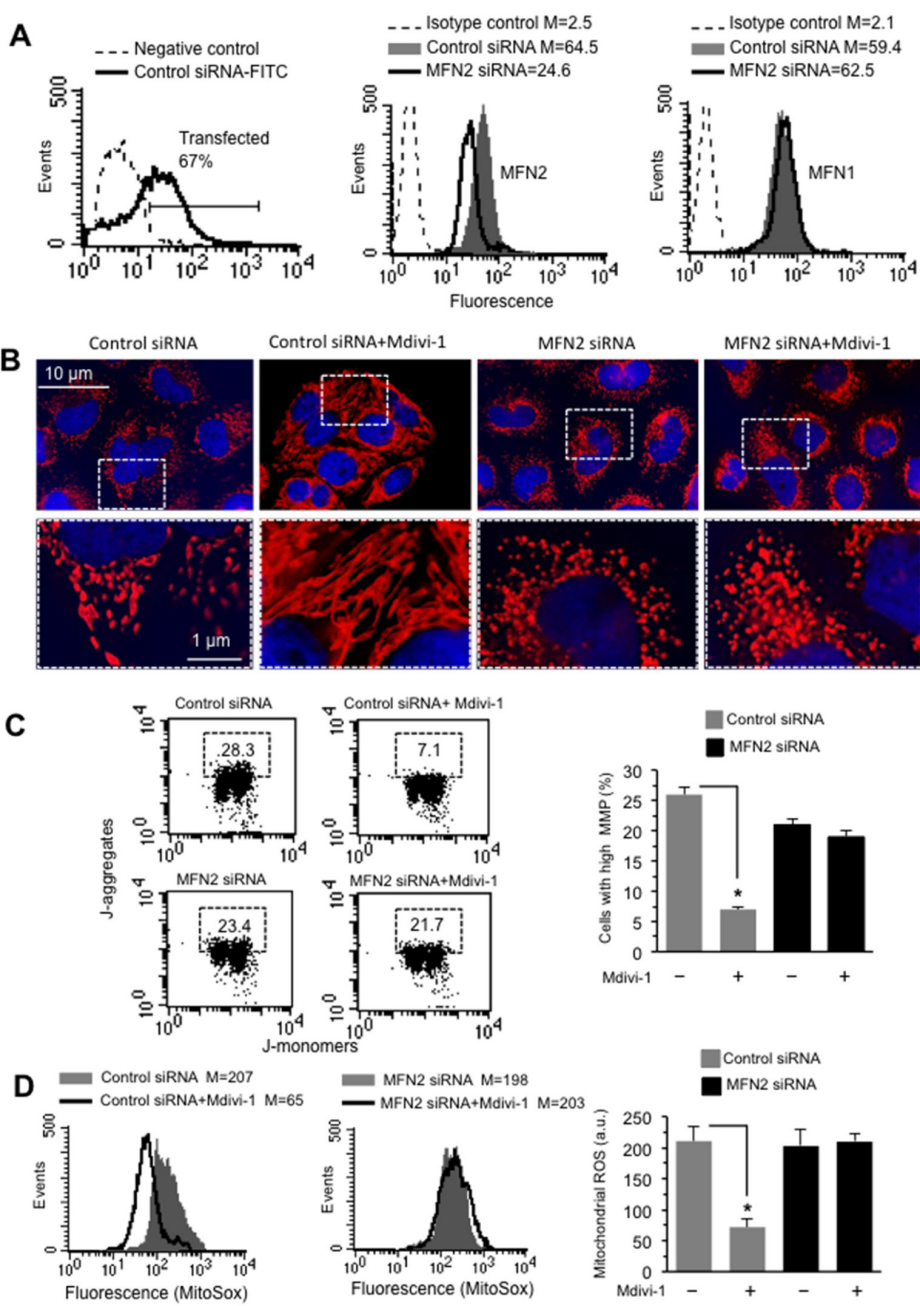
Effect of MF2 silencing on mitochondria network organization (**A**) *Left panel*. Flow cytometry evaluation of fluorescence in HeLa cells transfected with FITC-siRNA. The number in the left panel represents the percentage of transfected cells. Cytofluorimetric evaluation, performed 48 h after transfection, of MFN2 (central panel), or MFN1 (right panel) in cells transfected with non-silencing siRNA (Control siRNA) or with MFN2 silencing siRNA. Numbers represent the median fluorescence intensity and indicate the expression level of MFN1 and MFN2. (**B**) *Upper line.* IVM analysis of mitochondrial network of HeLa cells transfected with non-silencing siRNA (Control siRNA) or with MFN2 silencing siRNA and treated or not with Mdivi-1 and stained with anti-mitochondrion (red) and counterstaining with Hoechst (blue). *Bottom line*. Magnification of the boxed areas indicated in the pictures shown in upper line. (**C**) *Left panels.* Dot plots of MMP flow cytometry analysis, performed by using JC-1, of HeLa cells transfected with non-silencing siRNA (Control siRNA) or with MFN2 silencing siRNA and treated with Mdivi-1. Numbers in the boxed areas indicate the percentage of cells with high MMP. *Right panel.* Bar graph showing the results of the analysis of MMP obtained from three independent experiments and reported as mean ± SD. ^*^p < 0.01 *vs* CTR. (**D**) *Left panels*. Flow cytometric analysis of mitochondrial ROS production by using MitoSOX-red of HeLa cells transfected with non-silencing siRNA (Control siRNA) or with MFN2 silencing siRNA and treated with Mdivi-1. Numbers represent the median fluorescence intensity. Results obtained in a representative experiment among three are shown. *Right panel*. Bar graph showing the results obtained from three independent experiments and reported as mean ± SD. Numbers indicate the median fluorescence intensity. (^*^) Indicates *p <* 0.01.

To verify the pivotal role of MFN2 on mitochondrial network organization we transfected cells with MFN2 siRNA before Mdivi-1 treatment. Under our experimental conditions, we found that: (i) about 70% of HeLa cells were transfected (Figure 6A, left panel); (ii) in cells transfected with MFN2 siRNA a significant reduction (about 67%) of MFN2 expression, with respect to non-silencing siRNA-transfected cells, was detectable (Figure 6A, central panel); and that (iii) MFN2 siRNA was actually specific for this protein. In fact, transfection of cells with MFN2 siRNA did not interfere with MFN1 expression (Figure 6A, right panel). Analysis of mitochondrial network organization, performed after immunofluorescence cell staining, clearly indicated that Mdivi-1 was able to increase mitochondria network in cells transfected with non-silencing siRNA, but not in MFN2 siRNA-transfected cells (Figure 6B). Importantly, we also observed that silencing MFN2 was capable of inducing *per se* mitochondrial network fragmentation (compare third panel with first panel in Figure 6B). This is better appreciable by observing micrographs at higher magnification (Figure 6B, bottom line). Very interestingly, and according with the above data, MFN2 siRNA was also able to prevent either mitochondrial membrane potential changes (Figure 6C, left panel) or mitochondrial ROS reduction (Figure 6D, left panel) associated with Mdivi-1 treatment. These results were also confirmed by quantitative analyses, performed pooling together data obtained from three independent experiments (Figure 6C and 6D, bar graph in the right panels). These results suggest that the recruitment of MFN2 (but not of MFN1) to mitochondrial raft-like microdomains could be mandatory to the fulfillment of Mdivi-1-induced mitochondrial network development.

## DISCUSSION

**Figure 7:**
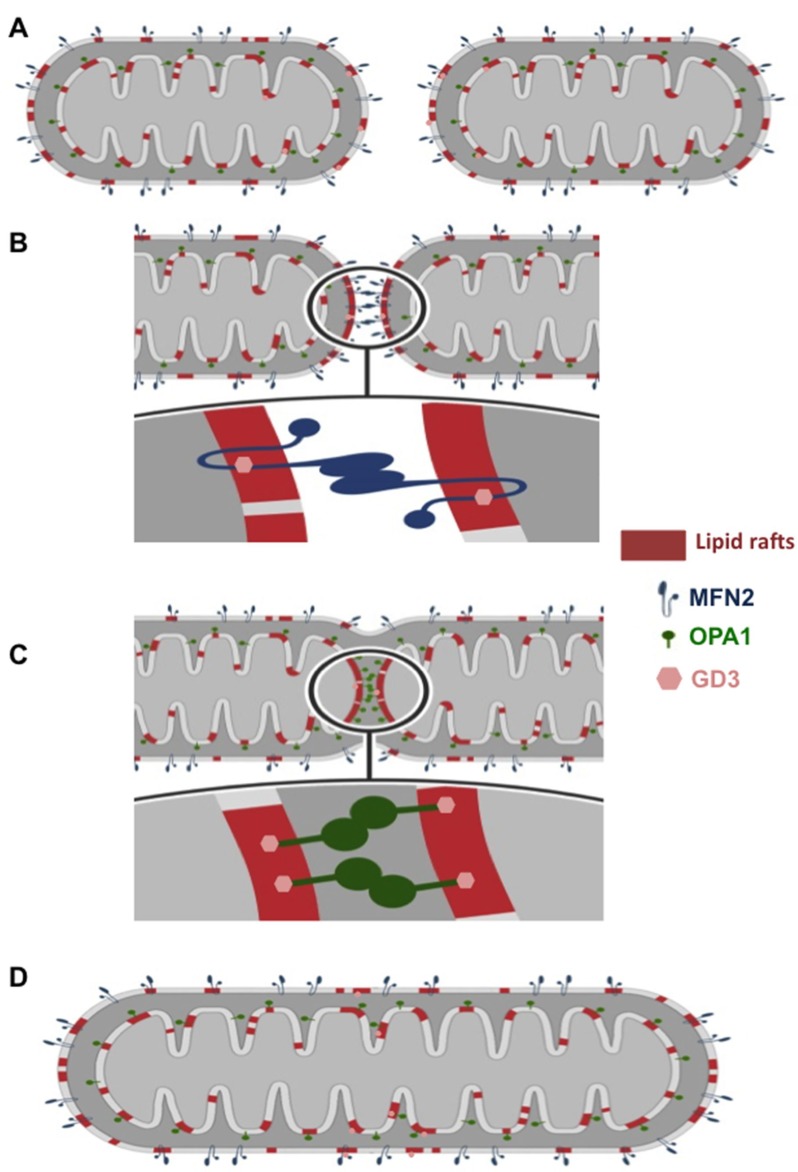
Cartoon schematizing mitochondria fusion process (**A**) Two mitochondria come close enough to interact. During the early stages of interaction, their outer membranes (OMs) and their inner membranes (IMs) reorganize to include the proteins necessary for fusion within raft-like microdomains; (**B**) OMs of two mitochondria fuse; (**C**) IMs fuse; (**D**) the new mitochondrion, which results from the fusion, reorganizes its membranes by returning to the initial state. In our hypothesis OM fusion is mediated by MFN2, a dynamin-related GTPase at the OM. IM fusion is mediated by OPA1. “Lipid rafts” could act as a sort of signaling device catalyzing key critical reactions for fusion process induced by chemical inhibition of the GTPase activity of DLP1 by Mdivi-1. In this model we suggest the mandatory role of the molecular association of MFN2 or OPA1 with the glycosphingolipid GD3 within mitochondrial raft-like microdomains during this process.

In the present study we provide morphological, functional, and mechanistic evidence that localization of MFN2 in lipid rafts, *via* its molecular interaction with the ganglioside GD3, is mandatory in Mdivi-1-induced mitochondria network organization. In a previous work, we have demonstrated (either in HeLa cells or in lymphocytes) that, under apoptotic stimulation, the recruitment of fission-associated molecules to the mitochondrial-like microdomains (“lipid rafts”) could play a role in the morphogenetic changes leading to organelle fission [20]. Thus, based on the assumption that lipid rafts could act as a sort of signaling device catalyzing key critical reactions [28, 29], we hypothesized that mitochondrial raft-like microdomains could play a fundamental role also in the extension of mitochondrial interconnected network due to the chemical inhibition of the GTPase activity of DLP1 by Mdivi-1. To verify this hypothesis, we analyzed herein the expression, function and intracellular localization of one of the main proteins of the fusion machinery: MFN2, which mediates the fusion of outer mitochondrial membranes. Biochemical analyses performed after cell fractionation and quantitative evaluations carried out by FRET technique clearly suggested that recruitment of MFN2 into “lipid rafts” was necessary to trigger mitochondria fusion process after Mdivi-1 treatment. In particular, as outlined in the schematic cartoon (shown in Figure 7A and 7B), MFN2 promotes the fusion of the outer mitochondrial membranes only once embedded into lipid rafts. This hypothesis is supported by the detection of a molecular association of MFN2 with GD3, selected as paradigmatic lipid component of mitochondrial lipid rafts [30], as well as by the results obtained either by altering lipid rafts organization by FB1 or by knocking down MFN2 by specific siRNA.

The mitochondrial fusion and fission involve a variety of proteins, which assist the cell throughout the series of events that drive these specific processes. The most relevant proteins engaged in the mitochondrial fusion process are three GTPase dynamin-like proteins: mitofusin 1 (MFN1) and 2 (MFN2), located at the outer mitochondrial membrane, and optic atrophy protein 1 (OPA1), at the inner membrane [31]. In this paper, we show that OPA1 is also recruited into mitochondrial lipid rafts during Mdivi-1-induced mitochondrial network “extension”, helping to regulate mitochondria morphology by participating to the fusion of the inner membrane of mitochondria (see cartoon in Figure 7C and 7D). These data add another piece to the knowledge on the molecular mechanisms underlying the mitochondrial dynamics. In fact, although fusion is crucial for the health and physiological functions of mitochondria, including complementation of damaged mitochondrial DNAs and the maintenance of membrane potential, the molecular mechanisms of this process still remain substantially unknown. However, due to the presence of MAM-specific marker in our mitochondrial preparations such as FACL4, we cannot exclude the possibility of mitochondria-associated membranes (MAMs) implication, since they display the characteristics of raft-like microdomains [27].

Mitochondria may increase or decrease the susceptibility of a cell to apoptosis through different ways. If on a one hand mitochondrial functions appear very sensitive to oxidative stress, on the other hand a dysfunction of mitochondria induced itself an excessive generation of ROS, which activates the signaling of JNK, p53, and caspase-3 [32, 33] and generates more ROS, resulting in a feedback cycle leading to cell death [34].

According with literature data, our observations clearly indicated that treatment with Mdivi-1 before apoptotic triggering by STS attenuated mitochondrial ROS generation, preserved MMP, and significantly inhibited apoptotic cell death. Although it appears that mitochondrial fusion proteins are not essential components of the apoptotic pathway [35–37], the fusion process activated by Mdivi-1 seems able to hinder cell death. Thus, we can hypothesize that the fusion process could act indirectly by preventing mitochondrial alterations, such as membrane depolarization and permeability transition pore opening, which cause the release of cytochrome C and apoptosis-inducing factors involved in the apoptotic execution pathway [38–40].

The regulation of mitochondrial fission/fusion is central for cell fate and its defects are directly involved in numerous human pathologies, especially neurological [41, 42] and cardiac disorders [43]. In fact, an expanding number of degenerative disorders have been associated with mutations in the genes encoding MFN2 and OPA1, including the rare Charcot-Marie-Tooth disease type 2A and autosomal dominant optic atrophy, and they also play a significant role in the molecular and cellular pathogenesis of more common neurodegenerative diseases, including Alzheimer’s and Parkinson’s diseases [44–45]. Hence, the modulation of fission/fusion processes recently gained the attention of translational research and physicians. Furthermore, since it was observed that senescent mitochondria exhibit morphological and functional alterations similar to those observed in neurodegenerative diseases, the interest of fission and fusion processes have also met the interest of other research groups involved in the investigation on successful cell senescence and longevity [45]. According with this, we previously hypothesized that mitochondria hyperfusion observed in fibroblasts isolated from centenarian subjects, i.e. in cells from a selected population of subjects undergone successful and healthy aging, played a pivotal role in the maintenance of mitochondria energy supply in longevity [46]. Finally, according to the results reported in this paper, it has been proposed that mitochondria hyperfusion and formation of mitochondrial networks could render the cells more resistant to death and mitophagy during nutrient depletion [46, 47] by selectively blocking the autophagic degradation of mitochondria [12].

Thus, on the basis of literature data and on the present results, we can hypothesize that mitochondrial dynamics could represent a promising novel target for many diseases. In fact, although not yet discovered, small molecule activators of DLP1 and inhibitors of mitochondrial fusion can be attractive as anti-cancer therapies as they have the potential to stimulate apoptotic cell death. By contrast, drugs such as Mdivi-1, inhibiting mitochondrial division and apoptosis in mammalian cells can be of potentially great clinical significance in neurodegenerative and cardiovascular disorders [48] as well as in the successful healthy aging of the cell.

## MATERIALS AND METHODS

### Cells and treatments

Human HeLa cells were maintained in DMEM (Gibco-BRL, Life Technologies Italia, Milano, Italy), containing 10% Fetal Bovine Serum (FBS, Gibco-BRL) and 100 U/ml penicillin and 100 mg/ml streptomycin, (Gibco-BRL) at 37° C in a humified 5% CO_2_ atmosphere. To induce mitochondria tubulation, cells were treated with 3-(2,4-Dichloro-5-methoxyphenyl)-2,3-dihydro-2-thioxo-4(1H)-quinazolinone (Mdivi-1, ENZO Life Sciences, Lausen, Switzerland), a cell-permeable selective inhibitor of dynamin-related protein 1 (DRP1) for 30 min, 1 hour, 2 hours and 4 hours. To alter raft organization, cells were treated with 30 mM Fumonisin B1 (FB1, Sigma-Aldrich, St. Louis, MO, USA), an inhibitor of ceramide synthase, for 24 hours. To inhibit microtubule polymerization, cells were treated with 0.3 μg/ml Demecolcine for 18 hours (DMC, Sigma-Aldrich). After treatments (performed at 37° C in a humidified 5% CO_2_ atmosphere), cells were collected and prepared for other procedures, as described below.

### Cell death assays

Quantitative evaluation of apoptosis was performed by a double staining flow cytometry method using fluorescein isothiocyanate (FITC)-conjugated Annexin V/Trypan blue (TB) apoptosis detection kit (Marine Biological Laboratory, MBL, Woods Hole, MA, USA) according to the manufacturer’s protocol. Which allows discrimination among early apoptotic, late apoptotic and necrotic cells.

### Flow cytometry analysis of the mitochondrial ROS (Reacting Oxygen Species)

Cells (5 × 10^5^) were incubated with 5 μM MitoSOX (Red Mitochondrial Superoxide Indicator, Thermo Fisher, Eugene, OR) in complete medium, for 30 min at 37° C. Cells were then washed in PBS and immediately analyzed on a cytometer.

### Immunofluorescence analysis

Control and treated cells were fixed with 4% paraformaldehyde (Carlo Erba, Milano, Italia) and then permeabilized by 0.5% Triton X-100 (Sigma-Aldrich). After washings, cells were incubated with the following monoclonal (MAb) or polyclonal (PAb) antibodies, alone or in combination, for 1 hour at 4° C: MAb to mitochondria (Chemicon, Temecula, CA, USA); MAb anti-GD3 (Abcam, Cambridge, UK), PAb anti-MNF2 (Cell Signaling, New England Biolabs, UK), PAb anti-OPA1 (BD Biosciences, Qume Drive, San Jose, CA), and MAb anti-DLP1 (BD Biosciences). After washings, cells were incubated with anti-mouse AlexaFluor 488-conjugated or AlexaFluor 594-conjugated and anti-rabbit AlexaFluor 488-conjugated or AlexaFluor 594-conjugated (all Termo Fischer) for additional 45 min at 37° C. All samples were counterstained with Hoechst 33342 and mounted with glycerol-PBS (2:1). The images were acquired by intensified video microscopy (IVM) with an Olympus fluorescence microscope (Olympus Corporation of the Americas, Center Valley, PA.), equipped with a Zeiss charge-coupled device (CCD) camera (Carl Zeiss, Oberkochen, Germany).

### Morphometric analysis

Quantitative evaluations of the percentage of cells with hyperfused mitochondria was carried out by evaluating at least 70 cells at the same magnification (1,300×) by using IAS2000 software (Delta Sistemi, Milan, Italy). The analysis was performed by analyzing at least twenty different fluorescence images for each experimental point.

### Sucrose-gradient fractionation

Lipid raft fractions from HeLa cells were isolated as previously described [19]. Briefly, 2 × 10^8^ cells were suspended in 1 ml of lysis buffer, containing 1% Triton X-100, 10 mM Tris-HCl (pH 7.5), 150 mM NaCl, 5 mM EDTA, 1 mM NaVO4 and 75 U of aprotinin and allowed to stand for 20 min. The cell suspension was mechanically disrupted by Dounce homogenization (10 strokes). The lysate was centrifuged for 5 min at 1300 × g to remove nuclei and large cellular debris. The supernatant fraction (post-nuclear fraction) was subjected to sucrose density gradient centrifugation; that is, the fraction was mixed with an equal volume of 85% sucrose (w/v) in lysis buffer (10 mM Tris-HCl (pH 7.5), 150 mM NaCl, 5 mM EDTA). The resulting diluent was placed at the bottom of a linear sucrose gradient (5–30%) in the same buffer and centrifuged at 200000 × g for 16–18 h at 4° C in an SW41 rotor (Beckman Institute, Palo Alto, CA, USA). After centrifugation, the gradient was fractionated, and 11 fractions were collected starting from the top of the tube. All steps were carried out at 4° C. The fraction samples were loaded by volume.

Alternatively, mitochondria isolated from cells, either untreated or treated with Mdivi-1 30 min, alone or in combination with FB1, were detergent solubilized according to Skibbens [49]. Briefly, mitochondria were lysed with 1 ml of extraction buffer (25 mM HEPES (pH 7.5), 0.15 NaCl, 1% Triton X-100 and 100 kallikrein U/ml aprotinin) for 20 min on ice. Lysates were collected and centrifuged for 2 min in a Brinkmann microfuge at 12000 × rpm at 4° C. Supernatants containing Triton X-100 soluble material were collected; pellets were undertaken to a second centrifugation (30 s) to remove the remaining soluble material. The pellets were then solubilized in 100 ml buffer containing 50 mM Tris-HCl (pH 8.8), 5 mM EDTA and 1% SDS. DNA was sheared by passage through a 22-gauge needle. Both Triton X-100-soluble and -insoluble material were analyzed by western blot analysis. The fraction samples were loaded by volume.

### Immunoblotting analysis of fractions

All the fractions, obtained as reported above, were subjected to sodium-dodecyl sulphate polyacrilamide gel electrophoresis (SDS-PAGE). The proteins were electrophoretically transferred onto polyvinilidene difluoride (PVDF) membranes (Bio-Rad, Hercules, California, USA) and probed with: MAb anti-OPA1 (BD Biosciences), PAb anti-MFN2 (Cell Signaling), MAb anti-DLP1 (BD Biosciences), or PAb anti-Flotillin 2 (Santa Cruz, California, USA). Bound antibodies were visualized with horseradish peroxidase (HRP)-conjugated anti-rabbit IgG or anti-mouse IgG (Jackson ImmunoResearch Laboratories, Baltimore Pike West Grove, PA, USA) and immunoreactivity assessed by chemiluminescence reaction, using the ECL Western detection system (Millipor, Darmstadt, Germania). Densitometric scanning analysis was performed by Chemidoc (Bio-Rad).

### Dot-Blot analysis

Decrease of GD3 was verified by densitometric scanning analysis of GD3 from immuno-dot-blot analysis. Briefly, aliquots, obtained from crude mitochondrial fractions of treated or untreated cells with FB1, were spotted onto nitrocellulose strips, and analysed by dot blot according to Garofalo *et al.* [27], using IgG anti-GD3 R24 mAb (Abcam, U.K., final dilution 1:500) for 1 h at room temperature.

### Knock-down experiments by MFN2 siRNA

HeLa cells were seeded (20.000 cells per dish) in a 60 mm dish in DMEM containing serum and antibiotics. Twenty-four hours after seeding, HeLa cells were transfected with GeneSolution siRNA (Qiagen Sciences, Germantown, MD, USA), according to the manufacturer’s instructions, using 10 nM Smart pool siRNA MFN2 for 48 hours. Transfection efficiency was evaluated by flow cytometry in cells transfected with Qiagen’s positive silencing siRNA (FITC-siRNA). As specific control, cells were also transfected with of non-silencing siRNA (AllStars Negative Control). After 48 hours, the effect of transfection was verified by flow cytometry by using MFN1 or MFN2 PAb (Cell Signaling).

### Preparation of crude mitochondrial fractions

Control and treated cells were resuspended in Homo buffer (10 mM HEPES (pH 7.4), 1 mM Ethylene Glycol-bis(3-aminoethyl Ether)N,N,N’N’Tetracetic Acid, (EGTA) 0.1 M sucrose, 5% bovine serum albumin BSA, (Sigma-Aldrich) 1 mM PMSF and complete protease inhibitor cocktail (Roche, Indianapolis, IN, USA) for 10 min on ice. Cells were homogenized with a Teflon homogenizer with B-type pestle [50] for 10 min at 4° C to remove intact cells and nuclei. The supernatants were further centrifuged at 10 000 × g at 4° C for 10 min to precipitate the heavy membrane fractions (enriched in mitochondria). These fractions were then subjected to further standard differential centrifugation. Thus, each fraction of our preparation was then tested by staining with Mab anti-Rab-5 (Santa Cruz Biotechnology) or Pab anti-EEA1 (Sigma-Aldrich), both specific to the endolysosomal compartment antigens, Mab anti-GM130 (Santa Cruz Biotechnology) a specific antigen to Golgi vesicles, Pab anti-ACSL4 (FACL4) (Santa Cruz Biotechnology) specific to MAMs, microsome or peroxisome, Pab TOMM20 (Santa Cruz Biotecnology) specific to a mitochondrial protein, or anti-CANX (Sigma-Aldrich) specific to Ca2+-binding ER palmitoylated chaperone protein enriched in the MAMs and in the endoplasmic reticulum.

### Mitochondrial membrane potential in living cells

The mitochondrial membrane potential (MMP) of controls and treated cells was studied by using 5-5′,6-6′-tetrachloro-1,1′,3,3′-tetraethylbenzimidazol-carbocyanine iodide probe (JC-1, Molecular Probes, Eugene, Oregon, USA). In line with this method, living cells were stained with 10 μM of JC-1. Tetramethylrhodamine ester 1 μM (TMRM, Molecular Probes) was also used to confirm data obtained by JC-1.

### Fluorescence resonance energy transfer by flow cytometry

We applied fluorescence resonance energy transfer (FRET) analysis by flow cytometry in order to study the molecular association of GD3 with MFN2, OPA1 or DLP1. The following primary antibody were used: MAb anti-GD3 directly conjugated with PE (Santa Cruz Biothechnology), PAb anti-MNF2 (Cell Signaling), PAb anti-OPA1 (BD Biosciences) and MAb anti-DLP1 (BD Biosciences).

Briefly, cells were fixed and permeabilized as above, washed twice in cold PBS and then labeled with antibodies tagged with donor (Phycoerythrin, PE) or acceptor (Cy5) dyes. In a second step, primary antibodies were detected by using specific secondary antibodies Cy5-conjugated anti-mouse or anti-rabbit. After staining, cells were washed twice, resuspended in PBS and analyzed with a dual-laser FACScalibur flow cytometer (BD Biosciences Franklin Lakes, New Jersey). For determination of FRET efficiency (FE), changes in fluorescence intensities of donor plus acceptor labeled cells were compared to the emission signal from cells labeled with donor-only and acceptor-only fluorophores. As a further control, the cross-reactivity among all the different primary and secondary antibodies was also assessed

Efficient energy transfer resulted in an increased acceptor emission on cells stained with both donor and acceptor dyes. The FRET efficiency (FE) was calculated according to Riemann [25] by using the following algorithm: FE = [FL3DA – FL2DA/a – FL4DA/b]/FL3DA, in which A is the acceptor and D the donor and where a = FL2D/FL3D and b = FL4A/FL3A. Fluorescence emission in channels 2 (PE), 3 (FRET) and 4 (Cy5) was expressed as median fluorescence.

### Data analysis and statistics

For morphometric studies, statistical analyses were performed by using Student’s *t* test (StatView for Macintosh; SAS Institute, Cary, NC, USA). For flow cytometry studies, all samples were analyzed with a FACScalibur cytometer (BD Biosciences) equipped with a 488 argon laser and with a 635 red diode laser. At least 30,000 events were acquired. Data were recorded and statistically analyzed by a Macintosh computer using CellQuest software (BD Biosciences). The expression level of the analyzed proteins on entire cells or isolated mitochondria was expressed as median fluorescence. Collected data analysis was carried out by ANOVA 2-way testing for repeated samples, using Graph Pad software (Graph Pad, San Diego, CA, USA). All data were verified in at least 3 independent experiments and are reported as means ± standard deviation (SD). Only values of *p <* 0.01 were considered as significant.

## SUPPLEMENTARY MATERIALS FIGURES


