# Supplementary material for: Recruitment of mitofusin 2 into “lipid rafts” drives mitochondria fusion induced by Mdivi-1
Source: Oncotarget. 2018 Apr 10;9(27):18869–84. doi: 10.18632/oncotarget.24792 (PMC5922362; doi:10.18632/oncotarget.24792)
Supplement: Supplementary file 1 [file oncotarget-09-18869-s001.pdf]

Recruitment of mitofusin 2 into "lipid rafts" drives mitochondria fusion induced by Mdivi-1

SUPPLEMENTARY MATERIALS

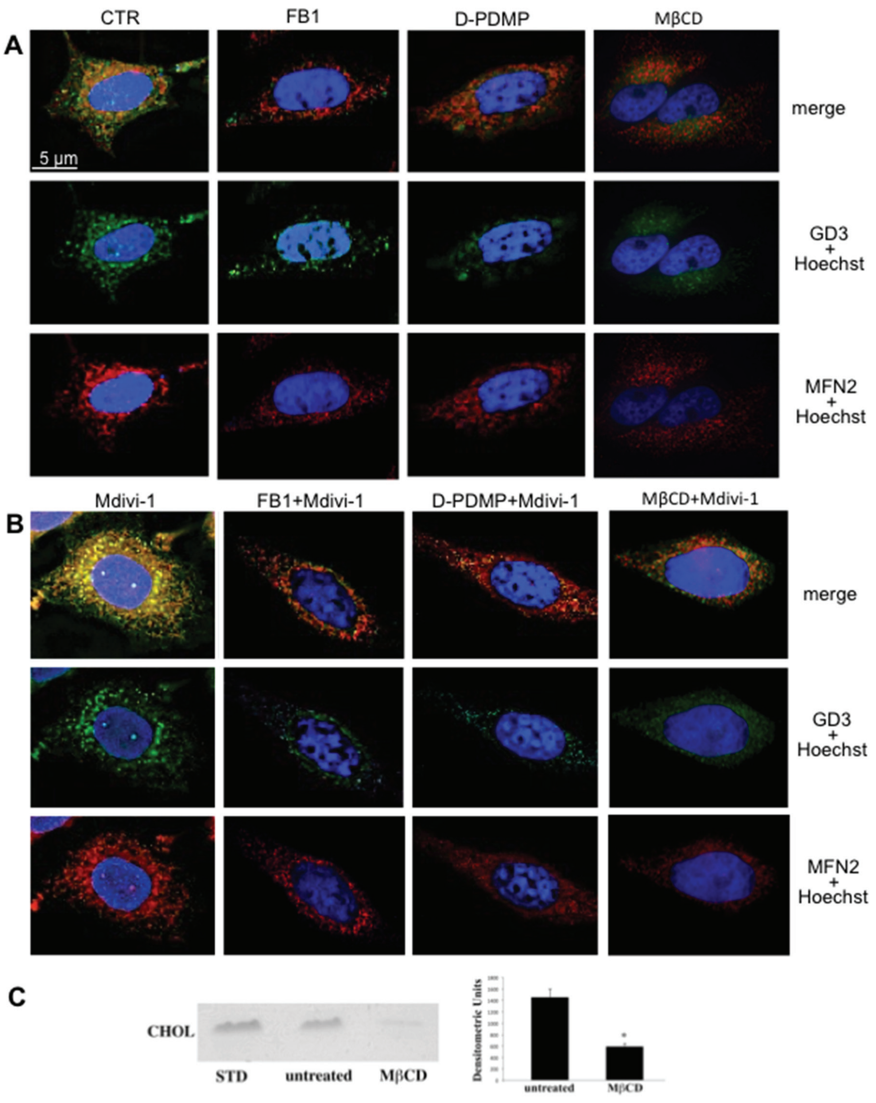

Supplementary Figure 1: (**A**) Immunofluorescence analysis of HeLa cells untreated, treated with FB1, D-PDMP or MbCD after staining with anti-GD3 (green), anti-MFN2 (red) and counterstaining with Hoechst (blue). (**B**) Immunofluorescence analysis of HeLa cells treated 30 min with Mdivi-1, alone or in combination with FB1, D-PDMP or MbCD, and stained with anti-GD3 (green), anti-MFN2 (red) and counterstaining with Hoechst (blue). (**C**) TLC analysis of total cholesterol (CHOL) from untreated or 5mM MbCD pre-treated cells; densitometric scanning analysis of cholesterol from TLC plates. Results represent the mean ± SD from 3 independent experiments, $^{*}P <$ 0.01 MbCD treated cells vs untreated cells.

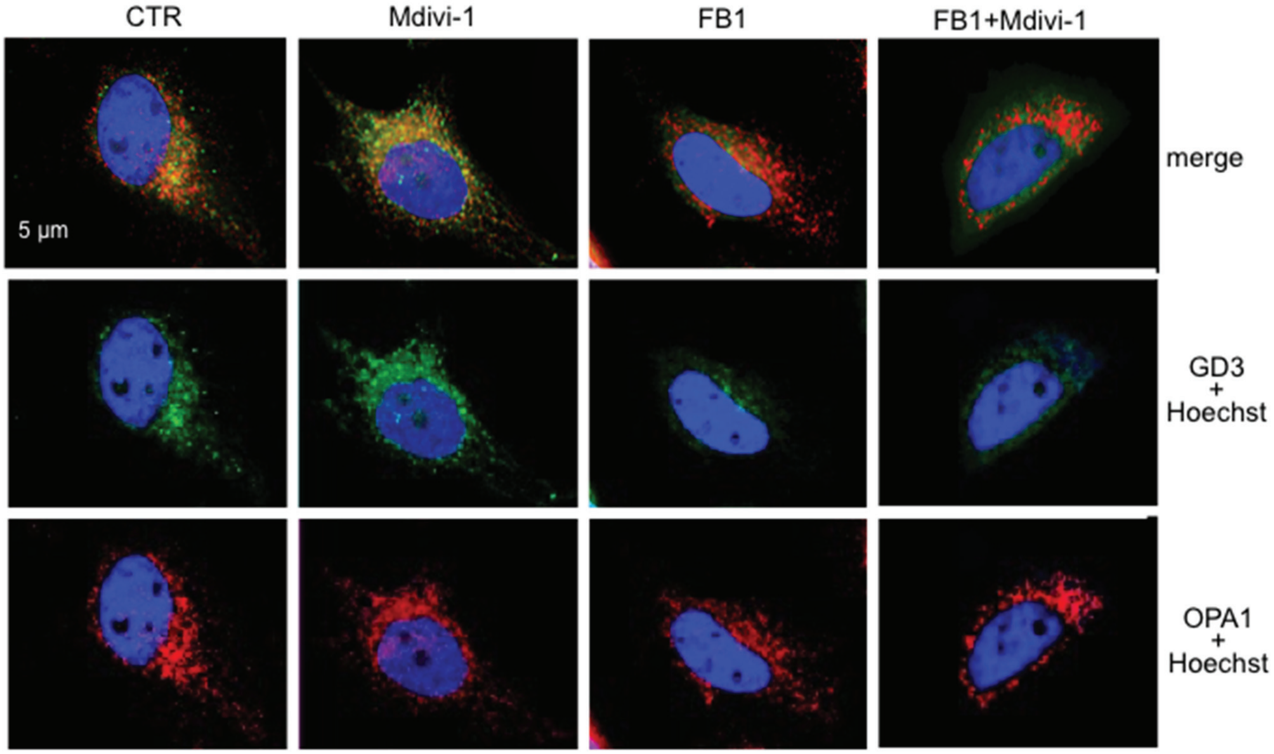

Supplementary Figure 2: Immunofluorescence analysis of HeLa cells untreated or treated 30 min with Mdivi-1 with or without FB1 after staining with anti-GD3 (green), anti-OPA1 (red) and counterstaining with Hoechst (blue).

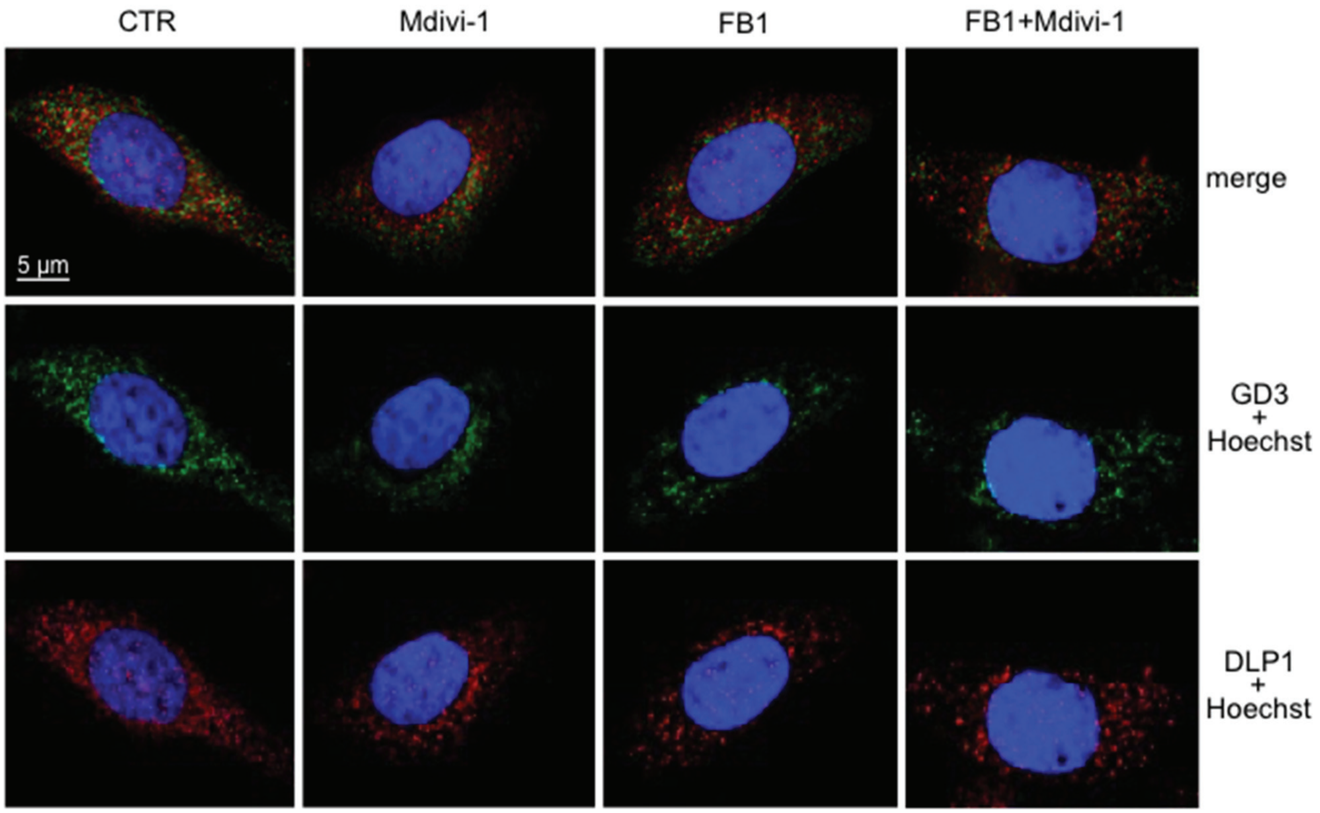

Supplementary Figure 3: Immunofluorescence analysis of HeLa cells untreated or treated 30 min with Mdivi-1 with or without FB1 after staining with anti-GD3 (green), anti-DLP1 (red) and counterstaining with Hoechst (blue).
